# Development and validation of nomograms by radiomic features on ultrasound imaging for predicting overall survival in patients with primary nodal diffuse large B-cell lymphoma

**DOI:** 10.3389/fonc.2022.991948

**Published:** 2022-12-07

**Authors:** Hongyan Deng, Yasu Zhou, Wenjuan Lu, Wenqin Chen, Ya Yuan, Lu Li, Hua Shu, Pingyang Zhang, Xinhua Ye

**Affiliations:** ^1^ Department of Ultrasound, The First Affiliated Hospital of Nanjing Medical University, Nanjing, Jiangsu, China; ^2^ Department of Cardiovascular Ultrasound, Nanjing First Hospital, Nanjing Medical University, Nanjing, Jiangsu, China

**Keywords:** diffuse large B-cell lymphoma, overall survival, radiomic, nomograms, predict

## Abstract

**Objectives:**

To develop and validate a nomogram to predict the overall survival (OS) of patients with primary nodal diffuse large B-cell lymphoma(N-DLBCL) based on radiomic features and clinical features.

**Materials and methods:**

A retrospective analysis was performed on 145 patients confirmed with N-DLBCL and they were randomly assigned to training set(n=78), internal validation set(n=33), external validation set(n=34). First, a clinical model (model 1) was established according to clinical features and ultrasound (US) results. Then, based on the radiomics features extracted from conventional ultrasound images, a radiomic signature was constructed (model 2), and the radiomics score (Rad-Score) was calculated. Finally, a comprehensive model was established (model 3) combined with Rad-score and clinical features. Receiver operating characteristic (ROC) curves were employed to evaluate the performance of model 1, model 2 and model 3. Based on model 3, we plotted a nomogram. Calibration curves were used to test the effectiveness of the nomogram, and decision curve analysis (DCA) was used to asset the nomogram in clinical use.

**Results:**

According to multivariate analysis, 3 clinical features and Rad-score were finally selected to construct the model 3, which showed better predictive value for OS in patients with N-DLBCL than mode 1 and model 2 in training (AUC,0. 891 vs. 0.779 vs.0.756), internal validation (AUC, 0.868 vs. 0.713, vs.0.756) and external validation (AUC, 914 vs. 0.866, vs.0.789) sets. Decision curve analysis demonstrated that the nomogram based on model 3 was more clinically useful than the other two models.

**Conclusion:**

The developed nomogram is a useful tool for precisely analyzing the prognosis of N-DLBCL patients, which could help clinicians in making personalized survival predictions and assessing individualized clinical options.

## Introduction

Diffuse large B-cell lymphoma (DLBCL) is the most common subtype of lymphoma, accounting for about 30% to 40% of the total incidence of all non-Hodgkin’s lymphomas (NHL) (1, 2). According to the site of origin, DLBCL can be divided into primary nodal diffuse large B-cell lymphoma (N-DLBCL) and primary extranodal diffuse large B-cell lymphoma (EN-DLBCL) (3). DLBCL has obvious heterogeneity in morphology, immunophenotype, genetics and clinical manifestations (4). Nowadays, the treatment of DLBCL has made great progress, immunochemical therapy of rituximab, cyclophosphamide, doxorubicin, vincristine and prednisone (R-CHOP) is the preferred treatment regimen for DLBCL (5). The application of rituximab increased the 5-year survival rate of DLBCL by at least 15% and the cure rate significantly, but there were still more than 30% of patients with primary drug resistance or relapse (6). These recurrent or refractory patients had a poor prognosis and high mortality (7). How to identify these patients as early as possible, accurately predict their efficacy and prognosis, and carry out individualized treatment? It has been an urgent clinical problem to be solved.

At present, 18F-deoxyglucose (FDG) positron emission tomography/computed tomography (PET/CT) has been widely used in evaluating the prognosis of DLBCL (8). Several studies (9, 10) have tested the use of metabolic intensity to predict Progression-Free Survival (PFS) and overall survival (OS) in patients with lymphoma. The most used parameter is the maximum standard uptake value (SUVmax) because it provides a method of measurement independent of the observer (11). However, the reliability of SUVmax may be affected by many factors, such as the attenuation of injection dose, the time between injection and imaging acquisition, partial volume effect and technical characteristics and parameters (12). Recently, new indicators for estimating the overall tumor load based on PET/CT staging, such as metabolic tumor volume (MTV) or total lesion glycolysis (TLG), have been used to predict PFS and OS in patients with lymphoma (13). In addition, international prognostic index (IPI) is currently used for estimating pretreatment risk, while IPI only comes from the clinicopathological features before treatment, which lacks the information to reflect the functional and metabolic characteristics of the tumor. Hence, the IPI often does not reliably predict the individual patient outcome (14).Therefore, the above evaluation indicators fail to capture the heterogeneity of tumors, which is a key prognostic factor for the progression, recurrence, and drug resistance of DLBCL, and closely related to tumor invasiveness, metastasis, and molecular characteristics. This limitation is a major challenge in DLBCL treatment. Therefore, we need to find a new imaging method, which can not only evaluate the treatment effect of patients in real time and dynamically, but capture the heterogeneity of metabolism in the tumor, to help clinicians modify the treatment plan in time and accurately predict the clinical outcome of DLBCL.

Ultrasound (US) can evaluate the shape, size, echo texture and blood flow pattern of lymph nodes in real time and dynamically (15). Radiomic is a method that uses complex computer algorithms to extract a large amount of data from images routinely obtained in the clinical environment, revealing hidden features of tumor from various imaging modes (16, 17), which can assist doctors to make the most accurate diagnosis by means of deeper mining and analysis of massive image data (18, 19). Hence, heterogeneity-related parameters provided by images could contribute to more personalized treatment and reduce the occurrence of toxicity. In this way, the possibility of favorable outcomes is increased, and intensive treatment programs can be provided for high-risk patients (20). Radiomic of 18F-FDG PET/CT have been demonstrated to be useful in predicting the outcomes of DLBCL and Hodgkin’s lymphoma (21, 22). Radiomic based on ultrasound has a good application prospect in the evaluation of curative effect and prognosis of other malignant tumors such as breast and gastrointestinal tumors (23–25). In lymphoma, radiomic shows hope in the differential diagnosis of lymphoma from other Lymph node diseases (26). To our knowledge, no previous study has associated radiomic signatures based on ultrasound with the outcome of patients with N-DLBCL.

This study was aimed at developing and validating the nomogram by radiomic features on ultrasound imaging to predict OS of patients with N-DLBCL more accurately and provide new ideas for personalized clinical treatment and visual evaluation of N-DLBCL.

### Patients

Patients newly confirmed with DLBCL and treated in our medical central from August 2009 to October 2021 were retrospectively analyzed. Exclusion criteria: ①Patients with other malignant tumors; ②Patients with Ann arbor IE staging; ③Patients treated in other hospitals or relapsed patients; ④Patients without complete US and clinical data. Inclusion criteria: ①Patients who underwent PET-CT; ②Lymph nodes with a maximum SUV value; ③Lymph nodes with core needle biopsy or resection biopsy. Finally, 111 patients with a total of 111 lymph nodes were included in this study. They were randomly assigned to training set(n=78) and internal validation set(n=33) (7:3 ratio). Besides, the independent external validation set consists of 34 patients from the other two institutions who meet the above exclusion and inclusion criteria. Histopathological diagnosis was based on the result of core needle biopsy or lymph node excision (excision biopsy is required only if the ultrasound-guided biopsy results are uncertain). Clinical variables of each patient were recorded, including gender, age at diagnosis, Bulky disease, B symptoms, Ann Arbor stage, Eastern Cooperative Oncology Group (ECOG), lactate dehydrogenase (LDH) level, serum β2-microglobulin (β2-MG) level, serum hemoglobin (HB) level, extra-nodal involvement, international prognostic index (IPI), state after first-line standardized chemotherapy, POD24, BCL6, BCL2 and treatment regimens. Disease staging was conducted in accordance with the Ann Arbor system. Bulky disease was defined as a nodal mass larger than 10 cm in diameter. State after first-line standardized chemotherapy were separated into two response categories as complete response(CR) and incomplete response (ICR, including partial response, stable and progression) (27).

The study was approved by the Institutional Ethics Committee of our hospital [Ethical number 2022-SR-058], and because it is a retrospective analysis, the requirement of written informed consent was waived.

### US image acquisition

The LogiqE9 ultrasound machine (GE Healthcare), with a 15–4 MHz linear probe (Super Linear™ SL15-4) was employed in ultrasonic examination. Two experienced radiologists used standardized institutional protocols to independently record and review all preoperative US features. If the radiologist had a different opinion on the conclusion, the final decision was made between them after a discussion. The patient was taken in a comfortable posture to fully expose the site of examination. The lymph node that underwent biopsy at the site of onset was selected as the target lymph node. The following parameters of lymph nodes were observed and measured, including size (cross-section, longitudinal-section), the ratio of the longitudinal diameter to the short axis (Solbiati index, SI>2, SI<2), sharp (regular, irregular), visibility of the hilum (present, absent), border (clear, unclear), Adler grade of blood flow (grades 0-3) (28).

### ROI segmentation and radiomics features extraction

The US images of all DLBCL patients were export from the Ultrasonic instrument. The maximum longitudinal-section area of images was manually segmented by two ultrasound experts (more than 5 years of experience) using open-source software (ITK-SNAP 3.8.0; http://www.itksnap.org) to generate a region of interest (ROI) containing all the segmented lesions. A total of 464 radiomics features were extracted from the US images, including 90 first-order features and 374 texture features. The first-order features include shape, size, and strength features and texture feature extraction is based on four texture matrices, including grey level cooccurrence matrix (GLCM), grey level run-length matrix (GLRLM), grey level size zone matrix (GLSZM) and gray level dependence matrix (GLDM). All radiomics features were analyzed and mined by PyRadiomic open-source software package.

### Radiomics features selection and signature calculation

One radiologist randomly selected 20 lesions from the training cohort to draw ROI again, and the other radiologist repeated it independently within three weeks. The stability of the feature is determined by calculating the inter-observe correlation coefficient (ICC). Radiomics features with ICC lower than 0.75 were excluded from the final feature data set (18).

To obtain the optimal subset of radiomics features, minimal redundancy maximum relevancy (mRMR) and the least absolute shrinkage selection operator (LASSO) with 10-fold cross-validation (the criteria as maximum area under the ROC curve) was further used to select most candidate radiomic features. Finally, 10 radiomics features were screened out. Therefore, the radiomics “Radcore” is calculated according to formula (1) (29).


(1) Radscore = β0 + β1χ1 +β2χ2 + … + βn χn

where β_0_ is the constant term in the regression, β_i_ is logistic regression coefficient, and χ_i_ the value of selected features.

### Construction of clinical model, radiomics model, and combined model

Univariate and multivariate COX regression analysis were used to analyze the influence of clinical variables, US characteristics. Then the characteristics of p< 0.05 in multivariate analysis were used to establish the model 1(clinical and US characteristics) of the training set. The optimal radiomics feature subset obtained by LASSO Logistic regression method was used to construct model 2 (radiomics features) in the training set. ROC curves were used to determine the cut-off value of each group feature, and the continuous variables were transformed into classified variables. The radiological signals constructed were mixed with clinical factors, and the univariate analysis and minimum Akaike information criterion (AIC) criterion COX regression analysis were obtained in turn. Model 3 (combined features) was constructed based on COX regression coefficient.

### Model performance assessment

Three established models were validated using independent internal and external datasets. The discriminant ability of each model was analyzed by receiver operating characteristic curve (ROC), and area under curve (AUC), sensitivity, specificity of them were obtained.

### Development and validation of the nomogram

According to the model 3, a nomogram, convenient for clinical application, was plotted. The model correction was evaluated by correction curve analysis and Hosmer-Lemeshow test. Decision curve analysis (DCA) was used to evaluate the clinical usefulness and net benefit of the predictive model in validation set. Delong test was used to compare the AUC of each pair of models.

### Patients’ treatment and follow-up

Patients diagnosed with N-DLBCL received standardized R-CHOP chemotherapy (n=105) or approved clinical drug verification(n=40). The follow-up data were obtained by electronic medical records and telephone interviews. Overall survival (OS) refers to the time from diagnosis until death due to any cause.

### Statistical analysis

The classification variables were expressed by the number of cases, using chi-square test (χ^2^) or Fisher exact test. A SPSS software (version 25.0) was used for univariate analysis and multivariate analysis. R software (version 3.6.1, R Project for Statistical Computing, www.r-project.org) was used for radiomic features analysis. The LASSO logistic regression method was implemented using the glmnet package in R software. Two-sided p value less than 0.05 was assumed to indicate statistical significance.

## Result

### Patient characteristics

**Figure 1:**
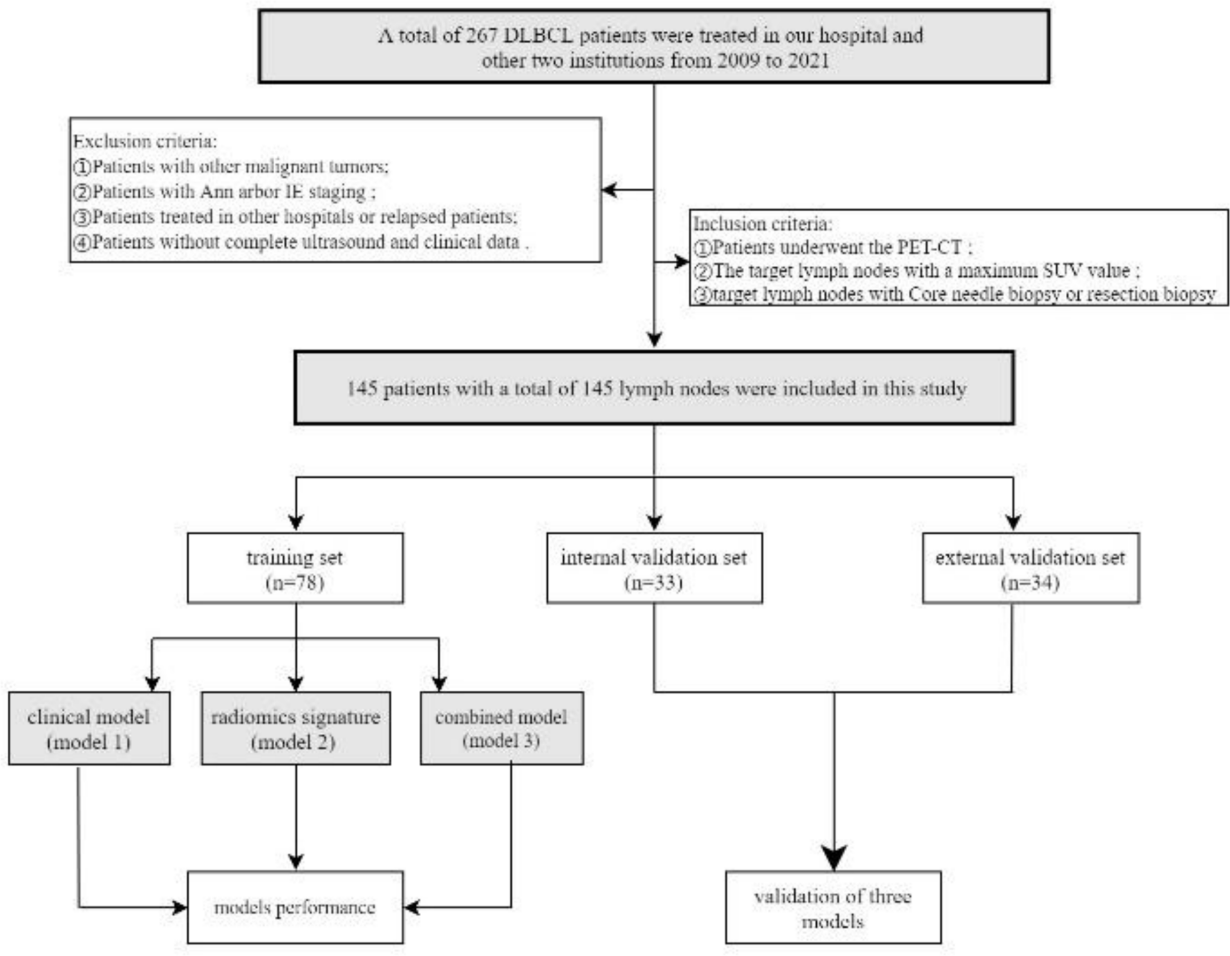
Flow chart of patients’recruitment pathway. DLBCL, Diffuse large B-cell lymphoma; PET-CT, Positron emission tomography/computed tomography. According to the inclusion and exclusion criteria, 145 patients with a total of 145 lymph nodes were included in this study. The patients of our institutions were randomly assigned to training set(n=78) and internal validation set(n=33) (7:3 ratio). The independent external validation set(n=34) from the other two institutions.

One hundred and forty-five patients with 145 lymph nodes were enrolled in this study, including 78 males and 67 females, ranging from the age of 21 to 85 (mean age, 58 ± 12). The research flowchart is shown in **Figure 1**. The median follow-up time was 36 months (range, 3–137 months). By the date of the last follow-up, a total of 41 patients had died, with a total survival rate of 71.7%. 1-year survival rate, 3-year survival rate and 5-year survival rate were 88.3%, 80.6% and 73.8% respectively. The clinical and ultrasonic features of the training set and verification set were summarized in **Table 1**. All the characteristics were not statistically significant between the two sets.

**Table 1:** Clinic and ultrasound features of training and validation sets.

Univariate analysis	Features	Training set(n=78)	Internal validation set(n=33)	p value	External validation set(n=33)	p value
	Gender			0.946		0.509
	Male	42(53.8)	18(54.5)		18(52.9)	
	Female	36(46.2)	15(45.5)		16(47.1)	
	age			0.982		0.681
	<60	41(52.5)	17(51.5)		16(47.1)	
	≥60	37(47.4)	16(48.5)		18(52.9)	
	Bulky			0.546		0.927
	<7.5cm	12(15.4)	3(9.1)		5(14.7)	
	≥7.6cm	66(84.6)	30(90.9)		29(85.3)	
	Extra-nodal involvement			0.285		0.235
	Yes	48(61.5)	18(54.5)		11(32.3)	
	No	30(38.5)	15(45.5)		23(67.6)	
	IPI			0.729		0.805
	0	37(47.4)	17(51.5)		15(44.1)	
	1-2	1(1.3)	1(3.0)		1(2.9)	
	3-5	40(51.3)	15(45.5)		18(52.9)	
	ECOG			0.946		0.235
	0-1	61(70.1)	25(75.8)		23(67.6)	
	≥2	16(29.9)	8(24.2)		11(32.4)	
	Stage			0.423		0.958
	I-II	18(23.1)	10(30.3)		8(23.5)	
	III-IV	60(76.9)	23(69.7)		26(76.5)	
	State of first			0.053		0.285
	CR	54(69.2)	16(48.5)		20(58.8)	
	ICR	24(30.8)	17(51.5)		14(41.2)	
	POD24			0.436		0.726
	No	62(79.5)	24(72.7)		28(82.4)	
	Yes	16(20.5)	9(27.3)		6(17/6)	
	LDH			0.577		0.656
	<271	31(39.7)	9(27.3)		6(18.2)	
	≥271	47(60.3)	24(72.7)		28(84.8)	
	b2mg			0.329		0.411
	<2.53	41(52.6)	14(42.4)		15(44.1)	
	≥2.53	37(47.4)	19(57.6)		19(55.9)	
	HB			0.628		0.592
	<120	37(47.4)	14(42.4)		12(35.3)	
	≥120	41(52.6)	19(57.6)		22(64.7)	
	BCL6			0.442		0.590
	−	17(21.8)	5(15.2)		9(26.5)	
	+	61(78.2)	28(84.8)		25(73.5)	
	BCL2			0.113		0.520
	−	18(23.1)	3(9.1)		6(17.6)	
	+	60(76.9)	30(90.9)		28(82.4)	
	SI			0.713		0.637
	SI≥2	51(65.4)	9(27.3)		12(35.3)	
	SI<2	27(34.6)	24(72.7)		22(64.7)	
	Hilus			0.215		0.745
	Absence	55(70.5)	25(75.8)		21(61.8)	
	Presence	23(29.5)	8(24.2)		13(38.2)	
	Border			0.886		0.631
	Clear	67(85.9)	24(72.7)		28(82.4)	
	Unclear	11(14.1)	9(27.3)		6(17.6)	
	Sharp			0.436		0.713
	Regular	51(65.4)	19(57.6)		21(61.8)	
	Irregular	27(34.6)	14(42.4)		13(38.2)	
	Alder			0.996		0.982
	0	20(25.6)	9(27.3)		8(23.5)	
	1	29(37.2)	13(39.4)		14(41.2)	
	2	15(19.2)	5(15.2)		6(17.6)	
	3	14(17.9)	6(18.2)		6(17.6)	

IPI, International Prognostic Index; ECOG, Eastern Cooperative Oncology Group; CR, Complete response; ICR, Incomplete response; LDH, Lactate dehydrogenase; HB, Hemoglobin; SI, Solbiati index, the ratio of the longitudinal diameter to the short axis.

### Model 1: Clinical features and US features

In the training set, univariate analysis in **Table 2** showed that five variables were related to OS. Multivariate analysis showed that hilus, extra-nodal involvement, state after first-line standardized chemotherapy were independent predictors of OS (p<0.05) (**Table 3**). The diagnostic performance of this model was moderate with an AUC of 0.779 (95% CI, 0.660-0.897). The sensitivity and specificity were 73.6% and 81.4%, respectively.

**Table 2:** Clinic and ultrasound features between survival group and death group.

Univariate analysis	Features	Training set (59/19)	p	Internal validation set (22/11)	p	External validation set (23/11)	p
	Gender		0.684		0.266		0.180
	Male	31(52.5)/11(57.9)		10(45.5)/8(72.7)		14(60.9)/4(36.4)	
	Female	28(47.5)/8(42.1)		12(54.5)/3(27.3)		9(39.1)/7(63.6)	
	age		0.695		0.325		0.897
	<60	31(52.5)/10(52.6)		10(45.5)/7(63.6)		11(47.8)/5(45.5)	
	≥60	28(47.5)/9(47.4)		12(54.5)/4(36.4)		12(52.2)/6(54.5)	
	Bulky		0.720		1.000		0.692
	<10cm	10(16.9)/2(10.5)		2(9.1)/1(9.1)		3(13.0)/2(18.2)	
	≥10cm	49(83.1)/17(89.5)		20(90.9)/10(90.9)		20(87.0)/9(81.8)	
	Extra-nodal involvement		0.006		0.034		0.016
	Yes	31(52.5)/17(89.5)		9(40.9)/9(81.8)		12(52.2)/1(9.1)	
	No	28(47.5)/2(10.5)		13(59.1)/2(18.2)		11(47.8)/10(90.9)	
	IPI		0.076		0.338		0.256
	0	32(54.2)/5(26.3)		15(68.1)/2(18.2)		12(52.2)/3(27.3)	
	1-2	1(1.7)/0(0.0)		1(4.5)/0(0.0)		1(4.3)/0(0.0)	
	3-5	26(44.1)/14(73.7)		6(27.2)/9(81.8)		10(43.5)/8(72.7)	
	ECOG		0.583		0.774		0.222
	0-1	47(79.7)/14(73.7)		17(77.3)/8(72.7)		14(60.9)/9(81.8)	
	≥2	12(20.3)/5(26.3)		5(22.7)/3(27.3)		9(39.1)/2(18.2)	
	Stage		0.211		0.430		0.611
	I-II	16(27.1)/2(10.5)		8(36.4)/2(18.2)		6(26.1)/2(18.2)	
	III-IV	43(72.9)/17(89.5)		14(63.6)/9(81.8)		17(73.9)/9(81.8)	
	State of first		0.001		0.002		0.023
	CR	47(79.7)/7(36.8)		15(68.2)/1(9.1)		17(73.9)/3(27.3)	
	ICR	12(20.3)/12(63.2)		7(31.8)/10(90.9)		6(26.1)/8(72.7)	
	POD24		0.043		0.002		0.048
	No	50(84.7)/12(63.2)		20(90.9)/4(36.4)		21(91.3)/7(63.6)	
	Yes	9(15.3)/7(36.8)		2(9.1)/7(63.6)		2(8.7)/4(36.4)	
	LDH		0.065		0.681		0.053
	<271	27(45.8)/4(21.1)		7(31.8)/2(18.2)		21(91.3)/7(63.6)	
	≥271	32(54.2)/15(78.9)		15(68.2)/9 (81.8)		2(8.7)/4(36.4)	
	b2mg		0.429		0.719		0.387
	<2.53	33(55.9)/8(42.1)		10(45.5)/4(36.4)		9(39.1)/6(54.5)	
	≥2.53	26(44.1)/11(57.9)		12(54.5)/7(63.6)		14(60.9)/5(45.5)	
	HB		0.186		0.136		0.245
	<120	25(42.4)/12(63.2)		7(31.8)/7(63.6)		6(26.0)/6(54.5)	
	≥120	34(57.6)/7(36.8)		15(68.2)/4(36.4)		17(73.9)/5(45.5)	
	BCL6		0.750		0.304		1.000
	−	12(20.3)/5(26.3)		2(9.1)/3(27.3)		6(26.1)/3(27.3)	
	+	47(79.7)/14(73.7)		20(90.9)/8(72.7)		17(73.9)/8(72.7)	
	BCL2		0.536		0.252		1.000
	−	15(25.4)/3(15.8)		1(4.5)/2(18.2)		4(17.4)/2(18.2)	
	+	44(74.6)/16(84.2)		21(95.5)/9(81.8)		19(82.6)/9(81.8)	
	SI		0.179		0.681		0.705
	SI≥2	41(69.5)/10(52.6)		7(31.8)/2(18.2)		9(39.1)/3(27.3)	
	SI<2	18(30.5)/9(47.4)		15(68.2)/9(81.8)		14(60.9)/8(72.7)	
	Hilus		0.045		0.012		0.016
	Absence	38(64.4)/17(89.5)		16(72.7)/9(81.8)		11(47.8)/10(90.9)	
	Presence	21(35.6)/2(10.5)		6(27.3)/2(18.2)		12(52.2)/1(9.1)	
	Border		0.012		0.033		0.048
	Clear	54(91.5)/13(68.4)		19(86.4)/5(45.5)		21(91.3)/7(63.6)	
	Unclear	5(8.5)/6(31.6)		3(13.6)/6(54.5)		2(8.7)/4(36.4)	
	Sharp		0.179		0.618		0.176
	Regular	41(69.5)/10(52.6)		12(54.5)/7(63.6)		15(69.6)/5(45.5)	
	Irregular	18(30.5)/9(47.4)		10(45.5)/4(36.4)		7(30.4)/6(54.5)	
	Alder		0.154		0.391		0.542
	0	13(22.0)/7(36.8)		7(31.8)/2(18.2)		5(21.7)/3(27.3)	
	1	20(33.9)/9(47.4)		8(36.4)/5(45.5)		8(34.8)/6(54.5)	
	2	14(23.7)/1(5.3)		2(9.1)/3(27.3)		5(21.7)/1(9.1)	
	3	12(20.3)/2(10.5)		5(22.7)/1(9.1)		5(21.7)/1(9.1)	

IPI, International Prognostic Index; ECOG, Eastern Cooperative Oncology Group; CR, Complete response; ICR, Incomplete response; LDH, Lactate dehydrogenase; HB, Hemoglobin; SI, Solbiati index, the ratio of the longitudinal diameter to the short axis.

**Table 3:** Multivariate analysis of clinical and ultrasonic features.

Multivariate analysis	Features	Estimate	Std.Error	Z value	p
Training set	Hilus	-2.369	1.161	-2.039	0.041
	Extra-nodal involvement	2.712	1.078	2.516	0.012
	State of first	0.986	0.482	2.045	0.041
Internal validation set	Hilus	-1.975	0.926	-2.132	<0.000
	Extra-nodal involvement	2.317	0.867	2.671	0.007
	State of first	1.201	0.404	2.973	0.003
External validation set	Hilus	-1.211	0.646	0.298	0.042
	Extra-nodal involvement	1.759	0.620	5.890	0.005
	State of first	1.454	0.491	4.28	0.003

### Model 2: Radiomics signature

**Figure 2:**
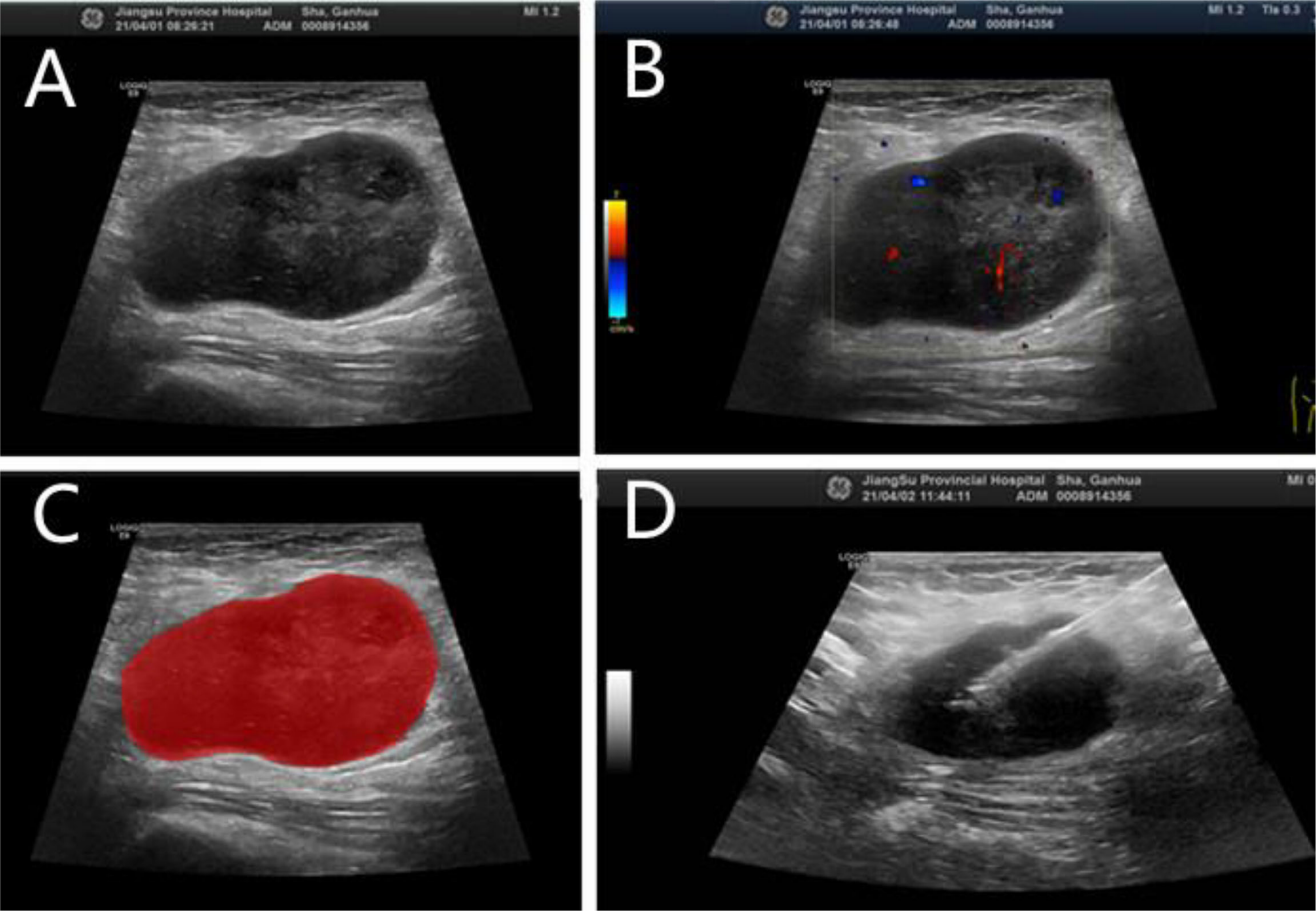
N-DLBCL in a 58-year-old man. **(A)** Gray-scale ultrasonic image. **(B**) CDFI image. **(C)** Radiomic ROI segmentation segmentation of the mass. **(D)** Ultrasound-guided core needle biopsy.

**Figure 3:**
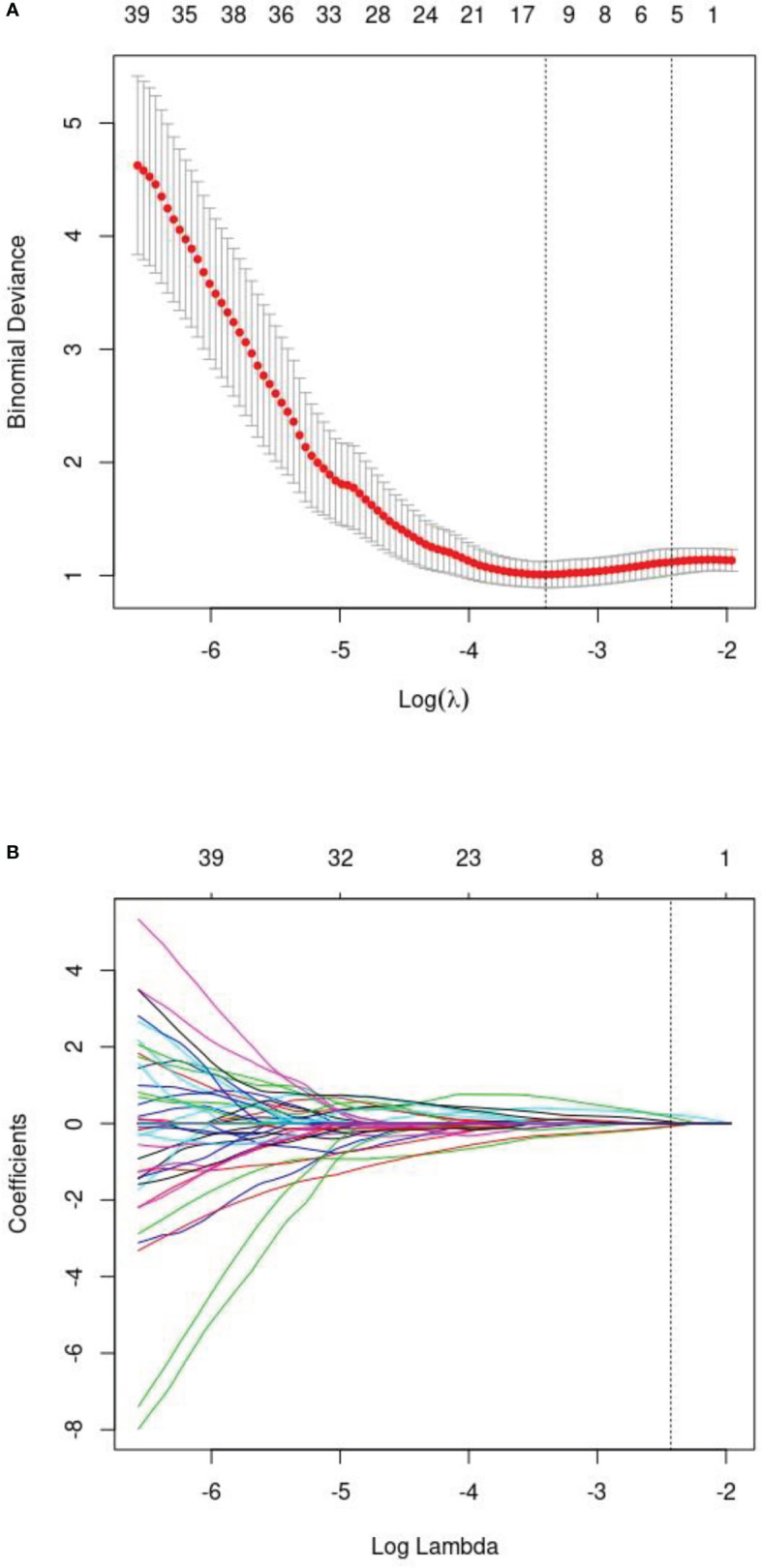
**(A)** The least absolute shrinkage andselection operator (LASSO) logistic regression for radiomics features selection and signature construction. **(B)** On the basis of minimum criteria for the least cross-validation binominal deviance, a tuning parameter (l) was selected via 10-fold cross validation.according to 10-fold cross-validation, 10 radiomic features were obtained.

After intra-observer and inter-observer reliability analysis, 340 stable features with ICC score larger than 0.75 were retained for follow-up analysis, and finally 10 radiomics features were selected into the LASSO Logistic regression model (**Figures 2**, **3**). ICC values are provided in the supplementary information. **Table 4** displays that variables A to J represent 10 selected radiomic features, Rad-score=−1.317+0.403×A+0.094×B+(−0.349) ×C+(−0.081) ×D+0.005×E+(−0.0.310) ×F+0.682×G+0.036×H+(−0.161) ×I+0.005×J. The discriminative ability of radiomics model was low with an AUC of 0.756 (95% CI, 0.622-0.889). The sensitivity and specificity are 93.2%, 52.6% respectively.

**Table 4:** Radiomics feature selection results.

Variables	Radiomics features name	Coef
A	original_glrlm_LowGrayLevelRunEmphasis	0.403277408
B	wavelet.LH_firstorder_Median	0.094560940
C	wavelet.LH_firstorder_Skewness	-0.349875709
D	wavelet.LH_glszm_LargeAreaHighGrayLevelEmphasis	-0.081542829
E	wavelet.LH_glszm_LargeAreaLowGrayLevelEmphasis	0.005653936
F	wavelet.HL_glcm_ClusterShad	-0.310516391
G	wavelet.HL_glszm_ZoneVariance	0.682458423
H	wavelet.HH_glcm_Idmn	0.035780343
I	wavelet.HH_glszm_GrayLevelNonUniformity	-0.161635402
J	wavelet.LL_ngtdm_Busyness	0.004573644

### Model 3: Comprehensive model

A comprehensive model was constructed based on multivariate Cox analysis of significant risk factors. The risk factors included Rad-score(p=0.012) and hilus (p=0.020), Extra-nodal involvement (p=0.027), state after first-line (p=0.023). The diagnostic efficiency of combined model is significantly improved, with an AUC of 0.891 (95% CI, 0.807-0.975). The sensitivity and specificity were 89.8% and 73.7% respectively.

### Validation and diagnostic performance comparison of three models

**Figure 4:**
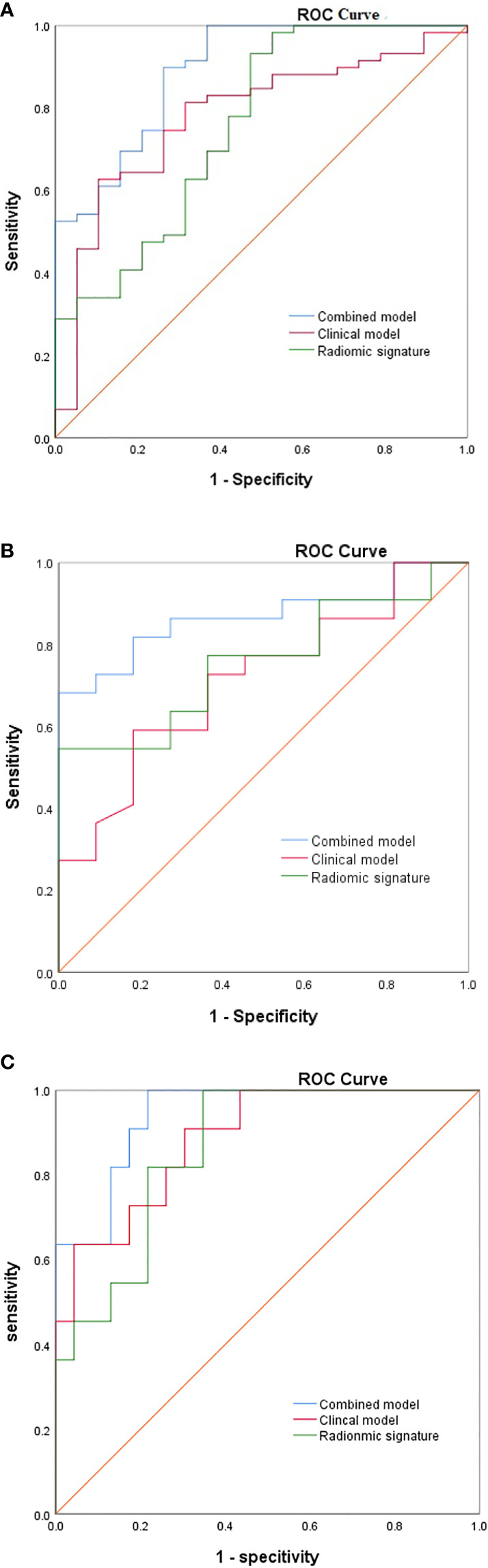
The ROC of the three model from **(A)** the training cohorts, **(B)** the internal validation cohort, **(C)** the external validation cohort.

Three established models were validated using independent datasets. In the internal validation set, the AUC, sensitivity, and specificity of model 1 were 0.713(95%CI,0.532-0.894), 59.1%, 81.8%. The AUC, sensitivity, specificity of model 2 were 0.756(95%CI,0.593-0.919), 54.5%, 73.2%; The AUC, sensitivity, specificity of model 3 were 0.868(95%CI,0.746-0.990), 81.8%, 81.8%. In the external validation set, the AUC, sensitivity, and specificity of model 1 were 0.866(95%CI,0.808-0.925), 87.7%, 80.8%. The AUC, sensitivity, specificity of model 2 were 0.789(95%CI,0.714-0.863), 92.7%, 60.6%; The AUC, sensitivity, specificity of model 3 were 0.914(95%CI,0.868-0.960), 95.1%, 81.7%. **Figure 4** shows the ROC curves of each model for both the training and validation sets, **Table 5** shows the cut-off value, sensitivity, specificity, and AUC of each mode. The AUC of the comprehensive model was significantly higher than that of model 1 or model 2 in both the training and validation sets.

**Table 5:** Diagnostic performance of three models.

Model	Cut-off value	AUC (95%CI)	Sensitivity %	Specificity %
Training set(n=78)				
Model 1	0.908	0.779(0.660-0.897)	73.6	81.4
Model 2	0.598	0.756(0.662-0.889)	93.2	52.6
Model 3	0.729	0.891(0.807-0.975)	89.8	73.3
Internal validation set (n=33)				
Model 1	0.891	0.713(0.532-0.894)	59.1	81.8
Model 2	0.932	0.756(0.593-0.919)	54.5	73.2
Model 3	0.843	0.868(0.746-0.990)	81.8	81.8
External validation set (n=34)				
Model 1	0.775	0.866(0.808-0.925)	87.7	80.8
Model 2	0.757	0.789(0.714-0.863)	92.7	60.6
Model 3	0.770	0.914(0.868-0.960)	95.1	81.7

### Development and performance of the nomogram

**Figure 5:**
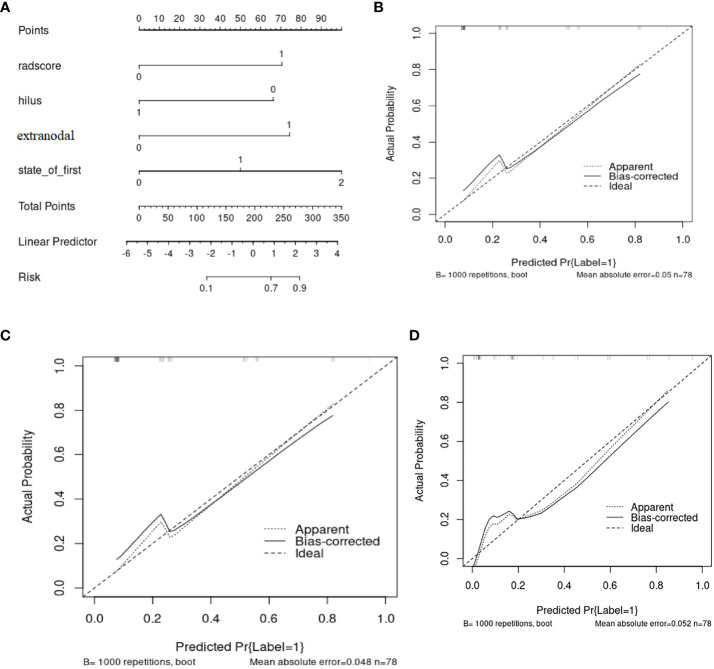
**(A)** The nomogram base on the model 3 and the calibration curve from **(B)** the training cohorts, **(C)** the internal validation cohort, **(D)** the internal validation cohort.

**Figure 6:**
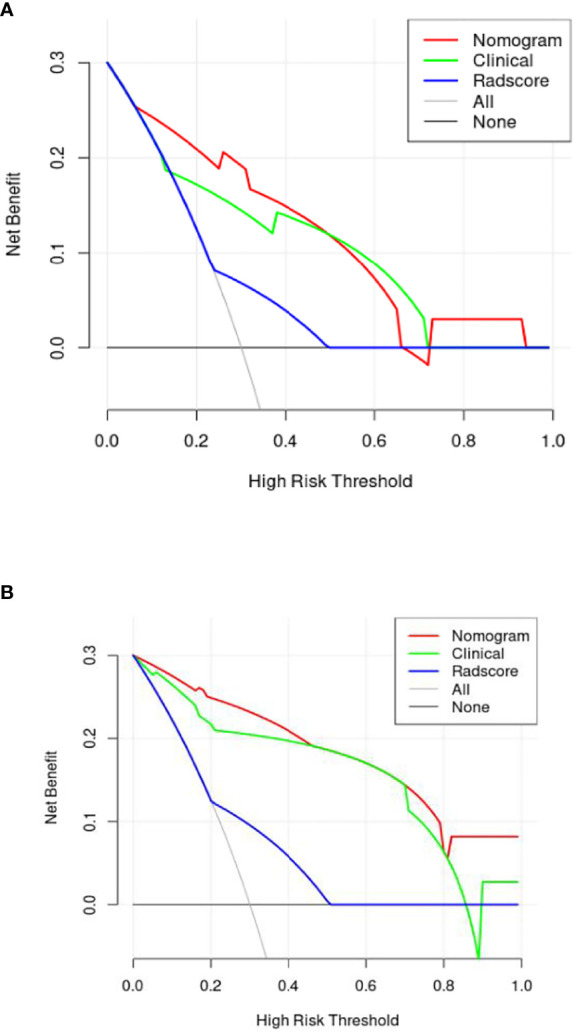
The decision curve [**(A)** is internal verification, **(B)** is external verification] analysis of the combined nomogram for prediction overall survival in patients with primary nodal Diffuse large B-cell lymphoma. The DCA indicated that the nomogram had a higher diagnostic efficiency than the clinical model and radiomics signature.

A nomogram (**Figure 5A**) was conducted based on the comprehensive model, and favorable calibrations of the nomogram were confirmed both in the training (**Figure 5B**), the internal validation set (**Figure 5C**), the internal validation set (**Figure 5D**). Hosmer–Lemeshow test possessed a p value of 0.334, 0.738 and 0.679, respectively. The DCA (**Figures 6A, B**) indicated that the nomogram had a higher diagnostic efficiency than model 1 or model 2.

## Discussion

This study established three models for predicting the prognosis of patients with N-DLBCL, and it was found that the model with the combination of radiomic and clinical features had good predictive value. On this basis, we developed a nomogram based on the combined model, and verified the nomogram. The results showed that the nomogram could well predict the OS of patients with N-DLBCL. Thus, nomograms can be used by clinicians to make precise and individualized medical decisions.

All N-DLBCL patients in our study were adults, which was in line with that reported in the literature for Chinese studies, Sun et al. (30)showed that the mean age of DLBCL patients was 51.6 years. Besides, consistent with the previous study (30, 31), most of the DLBCL cohort in this study was male patients, who accounted for 53.7% (78/145). The 5-year survival rate of 145patients in this study was 73.8% (107/145), which was almost the same as the report (74.8%) of Xia et al. (32), but higher than previous reports (43%-52%) (33). The reason is that the subjects in this study and Xia et al. are all primary intranodal DLBCL. Moo-Kon Song et al. (34) reported EN- DLBCL, such as originating from the gastric, intestinal tract and so on are even worse, especially in the non-GCB type. Some studies (3, 35) also have demonstrated that the involvement of the extranodal tissue may lead to a worse prognosis for patients with nodal lesions. The results of univariate and multivariate analysis showed state after the 6 cycles standardized treatment was the prediction factors of OS. After 6 cycles of standardized treatment, according to the results of PET-CT assessment, the CR rate of patients was 62.1% (90/145) in our study, which was lower than that reported by Ivan et al (36). At present, the effect of BCL-6 gene translocation on the prognosis of DLBCL patients is still controversial. Most scholars believe that the prognosis of BCL-6 positive expression is better, but some scholars hold the opposite view. Akyurek et al. (37) found that BCL-6 gene translocation can affect the OS of DLBCL patients(p=0.04), but there is no significant effect on PFS. This phenomenon is more significant in non-GCB DLBCL patients. In addition, foreign studies (38, 39) conclude that MYC/BCL2 co-expression in DLBCL is associated with an aggressive clinical course, which is more common in the ABC subtype, and contributes to the overall inferior prognosis of patients with ABC-DLBCL. However, in this study, probably due to the small sample size, BCL2 and BCL6 were not predictors of OS. For the same reason, the results of multivariate analysis showed that the other clinical features reported in the literature, including IPI score, LDH level, HB level, β2-MG level, Bulky disease, ECOG, Arbor stage, POD 24, were not associated with OS in the training group and the verification group.

US is also a commonly used diagnostic imaging technique, which possess a higher sensitivity in the detection of superficial enlarged lymph nodes. The feature of lymph nodes can be evaluated according to the hilum, shape, border, size, echo texture and blood flow pattern of lymph nodes (40). In this study, univariate analysis found that the lymphatic hilum structure and boundary is related to OS, while multivariate study found that the absence of lymphatic hilum is an independent predictor of OS. B lymphocytes mainly settle in the superficial cortex of the lymph nodes, some studies demonstrated that the abnormal lymphocytes of DLBLC in the early stage grew locally and did not invade the whole lymph node, but in the late stage, the abnormal lymphocytes infiltrated into the whole lymph node, resulting in the disappearance of the lymphatic hilum or eccentric and thin stripe under pressure (41). At the same time, studies have shown that patients with stage III-IV have a poor prognosis (3, 34). Therefore, in model 1, the absence of lymphatic hilum is a predictor of OS (training set, p=0.045; internal validation set, p=0.012; external validation set, p=0.016). From the previous literatures, in lymphoma, the disease frequently arises inside the lymph node, and (depending on the aggressiveness and natural history of the tumor) it may never reach the subcapsular area, or it may progress in a centrifugal fashion to invade the whole lymph node; in high-grade aggressive lymphomas, the neoplastic cells may even reach the lymph node from outside (as with metastasis) when the disease originates in another lymph node of the group and subsequently infiltrates the remaining nodes, the lesions fused with each other and the boundary was not clear (42), which may explain why in univariate analysis, the boundary is related to OS (training set, p=0.012; internal validation set, p=0.033; external validation set, p=0.048). The univariate and multivariate analysis showed that other ultrasonic features were not related to OS (p≥0.05).

18F-FDG PET/CT has been widely used for diagnosis, staging and response assessment in DLBCL (43). In recent years, there are also many literatures (12, 14, 44) to predict the prognosis of DLBCL based on 18F-FDG PET/CT baseline radiomic features. However, we have not seen a specific explanation about which target lymph nodes should be selected as the object of study, nor the criteria for selection. In this study, the target lymph nodes with the highest SUV value on PET-CT and ultrasound-guided pathological biopsy (**Figure 2**) were selected as the objects of study to reduce false positive and false negative and improve accuracy. As far as we know, there have been no radiomics based on ultrasound images to predict the prognosis of DLBCL, and the literatures on molecular imaging radiomics of DLBCL are also very limited. Parvez et al. (45) found that GLNGLSZM correlated with disease free survival, and that kurtosis correlated with OS. Aide et al. (4, 46) found that skewness of skeletal heterogeneity was a prognostic factor for PFS, and long-zone high gray level emphasis from GLSZM was a prognostic parameter for 2-year event-free survival. Meanwhile, Cottereau et al. (47) reported that the radiomic feature characterizing lesion dissemination was associated with PFS and OS. Our study found that radiomic features related to OS included two first-order features, two GLCM, four GLSZM, a GLSZM and a GLRLM, which is consistent with the above literature reports. In addition, Wang et al. (39) reported that radiomics are not superior to traditional imaging parameters. In our study, the diagnostic efficacy of radiomic signature (training set, AUC=0.756; internal validation set, AUC=0.756; external validation set, AUC=0.789) is lower than that of clinical model (training set, AUC=0.779; internal validation set, AUC=0.713; external validation set, AUC=0.866), but it can be used as a supplementary index of clinical model, and the diagnostic efficacy of combined model (training set, AUC=0.891; internal validation set, AUC=0.868; external validation set, AUC=0.914) is higher than radiomic signature or clinical model.

Still, there are some limitations in our study. First, this was a retrospective study, and the sample size was relatively small. But we have been collecting more cases and collaborating with other medical centers to expand the sample size, using external verification sets to further validate the nomogram. Second, the radiomic features were only extracted from gray-scale ultrasound images, and hopefully in the future, we can extract them from multimode ultrasound images such as elastography and contrast-enhanced ultrasound.

In conclusion, based on clinic and radiomic features, we have developed and validated a nomogram to predict OS of patients with N-DLBCL. The established nomograms can provide a visualized estimation of risk for each prognostic factor, to assist clinicians take personalized treatment for N-DLBCL patients and improve their prognosis.

## Data availability statement

The original contributions presented in the study are included in the article/**Supplementary Material**. Further inquiries can be directed to the corresponding authors.

## Ethics statement

The studies involving human participants were reviewed and approved by The study was approved by the Institutional Ethics Committee of the First Affiliated Hospital of Nanjing Medical University [Ethical number 2022-SR-058], and the requirement of written informed consent was waived. Written informed consent for participation was not required for this study in accordance with the national legislation and the institutional requirements.

## Author contributions

HD designed the study and wrote the manuscript. YY, WL, YZ and WC collected the data. LL and HS analyzed the data. HD, PZ, and XY evaluated US images and segmented lesions. All authors contributed to the article and approved the submitted version.
